# Recent Insights Into the Prognostic and Therapeutic Applications of Lysozymes

**DOI:** 10.3389/fphar.2021.767642

**Published:** 2021-12-03

**Authors:** Lin Jiang, Yunhe Li, Liye Wang, Jian Guo, Wei Liu, Guixian Meng, Lei Zhang, Miao Li, Lina Cong, Meiyan Sun

**Affiliations:** ^1^ College of Laboratory Medicine, Jilin Medical University, Jilin, China; ^2^ Department of Pharmacological and Pharmaceutical Sciences, College of Pharmacy, University of Houston, Houston, TX, United States; ^3^ Department of Neurosurgery, China-Japan Union Hospital, Jilin University, Changchun, China; ^4^ School of Biological Engineering, Dalian Polytechnic University, Dalian, China

**Keywords:** lysozyme, prognosis, therapy, cancer, hypertension, viral disease

## Abstract

Lysozymes are naturally occurring enzymes present in a variety of biological organisms, such as bacteria, fungi, and animal bodily secretions and tissues. It is also the main ingredient of many ethnomedicines. It is well known that lysozymes and lysozyme-like enzymes can be used as anti-bacterial agents by degrading bacterial cell wall peptidoglycan that leads to cell death, and can also inhibit fungi, yeasts, and viruses. In addition to its direct antimicrobial activity, lysozyme is also an important component of the innate immune system in most mammals. Increasing evidence has shown the immune-modulatory effects of lysozymes against infection and inflammation. More recently, studies have revealed the anti-cancer activities of lysozyme in multiple types of tumors, potentially through its immune-modulatory activities. In this review, we summarized the major functions and underlying mechanisms of lysozymes derived from animal and plant sources. We highlighted the therapeutic applications and recent advances of lysozymes in cancers, hypertension, and viral diseases, aiming toseeking alternative therapies for standard medical treatment bypassing side effects. We also evaluated the role of lysozyme as a promising cancer marker for prognosis to indicate the outcomes recurrence for patients.

## Introduction

Lysozyme, also known as muramidase or N-acetylmuramoyl-hydrolase, is an alkaline protease, which can hydrolyze mucopolysaccharide in pathogenic bacteria (Vanderkelen et al., 2011). Lysozyme could been secreted by many organs, such as in blood, liver, secretory fluid, urine, saliva, milk, and on the mucosal surface (Callewaert and Michiels, 2010; Lelouard et al., 2010). Lysozyme exists not only in animal tissues and secretions, such as macrophages, neutrophils and dendritic cells but also in microbial cells and plant secretions (Pipe, 1990). It can be divided into animal lysozyme, plant lysozyme, microbial lysozyme and phage lysozyme based on different sources (Ding et al., 2014). Animal lysozyme can be further divided into conventional type (c-type, vertebrates and insects, etc.), goose type (g-type, birds, etc.) (Canfield and McMurry, 1967) and i-type (insects Invertebrates and marine bivalves, etc.) (Bachali et al., 2004; Yue et al., 2011). At beginning, the lysoson in Bacillus subtilis found by Nicolle (Fleming, 1922) is the predecessor of lysozyme. A few years later, Fleming et al. extracted a kind of protein that could dissolve cell walls and bacteria from human body fluids and tears. It was named lysozyme since it can dissolve bacteria (Fleming, 1922). In 1965, Phillip analyzed the three-dimensional structure of lysozyme via X-ray diffraction and developed a model, which promoted the research of lysozyme (Kirby, 2001). And Abraham et al. isolated lysozyme crystals from egg protein, which started the research chapter of lysozyme (Kirby, 2001).

**FIGURE 1:**
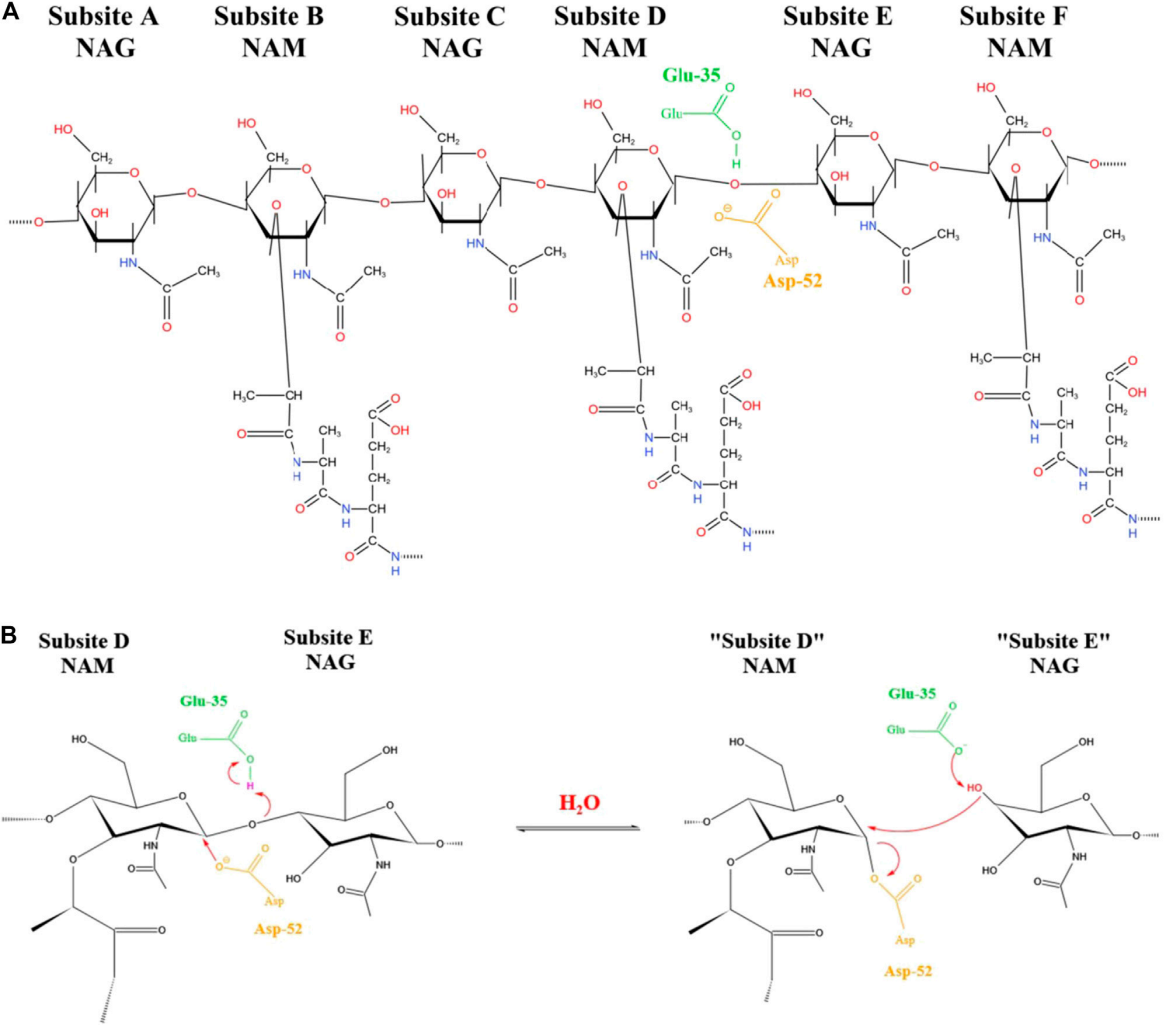
**Chemical structure simulation diagram of hydrolysis site of lysozyme**. The active site of lysozyme is bound to six continuous sugar monomers through six subsites (A-F), and then hydrolyzed by double substitution reaction β- 1,4 glycosidic bond, the catalytic group acts on the D site **(A)**. D and E are stretched into a half chair transition state, the catalytic group glutamic acid (Glu) 35 is bound to the D site, and aspartic acid (Asp) 52 is bound to the E site to hydrolyze the peptidoglycan skeleton of bacterial cell wall **(B)**.

Lysozyme is a well-known antibacterial polypeptide (Ganz, 2004; Nakatsuji and Gallo, 2012). It’s also the main effective ingredient of many ethnomedicines, including, *Pithecellobium dulce (Roxb.) Benth*[Leguminosae; *Pithecellobium dulce* seeds], and soft shelled turtle, sea cucumber recorded in *Compendium of Materia Medica* (Araki et al., 1998; Xia and Wang, 2015; Yang, 2015; Gálvez-Iriqui et al., 2020). The Supplement to the Compendium of Materia Medica in the Qing Dynasty reads, “The sea cucumber with black thorns produced in Liaodong is the best, which benefits to promote spermatogenesis, hematopoiesis and immunity” (Xia and Wang, 2015; Yang, 2015). Sea cucumber i-type lysozyme is the main component of sea cucumber to improve human immunity (Cong et al., 2009). It is found that the lysozyme in the seeds of *Pithecellobium Dulc*e, which is the main component, such as antifungal (Gálvez-Iriqui et al., 2020). As an important part of the innate immune system, lysozyme in human milk can protect infants and children from diarrhea (Cooper et al., 2013). In addition, lysozyme has been widely used in food, animal feed, medical device, and cosmetics (Cooper et al., 2013; Oliver and Wells, 2015; Zhang et al., 2021; Anastas et al., 2021). Lysozyme mainly breaks the β-1,4 glycosidic bond between N-acetylmuramic acid (NAM, MurNAc) and N-acetylglucosamine (NAG, GlcNAc) in the cell wall of bacterial (Turner et al., 2014). Functionally, it decomposes the insoluble mucopolysaccharide in the cell wall into soluble glycopeptides, as shown in Figure 1, resulting in the rupture of the cell wall that leads to the escape of the contents and the dead of bacteria (Ragland and Criss, 2017). It should be added that animal lysozymes, plant lysozymes, microbial lysozymes and phage lysozymes all hydrolyze similar polysaccharides (Gajda and Bugla-Ploskonska, 2014). These proteins do not show similarities in amino acid composition, but have a structurally stable core domain of “Helix- Link- Helix (HLH)” and two main catalytic groups of enzymes are Glu and Asp (Gajda and Bugla-Ploskonska, 2014). These enzymes represent a superfamily of hydrolases, which probably originated from a diversified evolution (Monzingo et al., 1996). HEWL and fungal lysozyme have the same core protein structure but contain different N-terminal and C-terminal domains (Monzingo et al., 1996). The decomposition activities of plant lysozyme to colloidal chitin is 10 times that of HEWL (Beintema and Terwisscha van Scheltinga, 1996). The primary structural difference between HEWL and human lysozyme is about 30%, so their tertiary structure is very different, which is determined by crystallography (Wilken and Nikolov, 2011). The differences in structure and biological activities of different types of lysozymes are summarized in Table 1. At present, it is mainly studied in HEWL and human lysozyme (Wilken and Nikolov, 2011). Emerging researches show evidence that lysozyme can not only directly resist bacteria, but also regulate the host immune response to infection (Sava, 1996). Lysozyme is called the cornerstone of biological innate immunity (Lönnerdal, 2003; Varahan et al., 2013). Studies have found that lysozyme is an integral part of the anti-bacterial pathway related to the monocyte-macrophage system (Osserman and Lawlor, 1966). Lysozyme functions by attacking, hydrolyzing, and breaking glycosidic bonds in peptidoglycans. The hydrolysate can enhance the secretion of immunoglobulin A (IgA), macrophage activation and rapid removal of bacterial pathogens (Kawano et al., 1981; Clarke et al., 2010). Large-scale production of HEWL can control the growth of sensitive bacteria and regulate the host’s immunity to infection and immune response inhibition (Sava, 1996). It is also reported that the levels of lysozyme indicates the risk of upper respiratory tract infection and is a biomarker of mucosal immune ability (Hanstock et al., 2019). It is considered to be the most promising antimicrobials that is able to be developed into a new clinical drug (Lepanto et al., 2019; Vilas Boas et al., 2019).

**TABLE 1:** The similarities and differences between animal- and plant-original lysozymes.

Type		Similarities (Physicochemical properties and Biological functions)	Differences		References
			Structure, physicochemical properties and biological function (sample)	Bacteriostatic spectrum	
Animal lysozyme	c-type	Physicochemical properties: Thermal stability:	Sample: HEWL	Gram (+)/Gram (−) (A small amount of lysozymes)	(Canfield and McMurry 1967; Howard and Glazer 1967, 1969; Barel et al., 1970; Inouye et al., 1970; Schoentgen et al., 1982; Beintema and Terwisscha van Scheltinga, 1996; Fastrez 1996; Bachali et al., 2004; Merlini and Bellotti 2005; Cong et al., 2009; Baase et al., 2010; Kajla et al., 2011; Wenzel et al., 2011; Yue et al., 2011; Airapetova et al., 2013; Li et al., 2018)
			Structure:		
		Strong thermal stability in acidic environment.			
			129 amino acid residues.		
		When pH 4–7, maintain its main biological activity after treatment at 100°C for 1 min			
			Molecular weight is 14KD.		
			Physicochemical properties:		
		When pH < 4, it can withstand 100°C treatment for 45 min.			
			pI 10.7–11.0.		
		Biological function:			
		Hydrolase activity (hydrolysis β- 1,4 glycosidic bonds).			
			Optimum temperature is 50°C.		
		Alkaline protease.			
		Non-specific immune factors:			
		non-specific immune factor	Biological function:		
		in the biological immune system.			
			The catalytic groups are located in Glu-35 and Asp-52.		
		can defend against the invasion of foreign microorganisms;			
	g-type		Sample: Goose egg white lysozyme	Gram (+)	
		All of them have a structurally stable core domain “Helix- Link- Helix (HLH)” and the two main catalytic groups of hydrolyze mucopolysaccharide are Glu and Asp.			
			Structure:		
			190 amino acid residues.		
			Molecular weight is 22 kD.		
			Physicochemical properties:		
			Optimum pH = 5.		
			Biological function:		
			Hydrolytic chitin activity (optimum pH =4.5).		
			The catalytic groups are located in Glu-71 and Asp-84.		
	i-type		Sample: i-type lysozyme of sea cucumber	Gram (+)/Gram (−)	
			Structure:		
			119 amino acids residues.		
			Molecular weight is 14.7 kD.		
			Physicochemical properties:		
			Optimum temperature is 35°C.		
			Optimum pH = 6.5.		
			Biological function:		
			The catalytic groups are located in Glu-14 and Asp-27.		
Plant lysozyme			Sample: papaya lysozyme	Gram (+)/Gram (−)	
			Structure:		
			212 amino acid residues.		
			Molecular weight is 25KD.		
			Physicochemical properties:		
			Optimum pH = 4.6.		
			Biological function:		
			Hydrolytic chitin activity.		
			The active catalytic groups are -COOH (Asp/Glu) and Cys.		
Microbial lysozyme	N-acetylhexosamine enzyme		Sample: N, O-diethylphthalide cytoplasmic enzyme	Gram (+)/Gram (−)	
	N-acety lmuramy-L-alanine amidase (MA)/Autolysin				
			Structure:		
			211 amino acids residues.		
	Endopeptidase		Molecular weight is 22.4 KD.		
			Physicochemical properties:		
			Optimum pH = 7.		
			Optimum temperature is 40°C.		
			Acid resistance.		
			Biological function:		
			The catalytic groups are located in Asp-6 and Glu-33.		
Phage lysozyme			Sample: phage T4 lysozyme	Gram (+)/Gram (−)	
			Structure:		
			164 amino acids residues.		
			Molecular weight is 18.7 KD.		
			Physicochemical properties:		
			Optimum temperature is 50		
			Optimum pH = 7.0.		
			Biological function:		
			The catalytic groups are located in Glu-11 and Asp-20.		

With the discovery of the immunomodulatory ability of lysozyme, lysozyme therapy has attracted extensive attention in the medical society. Its antiviral effect can be used together with immune stimulation to treat gastrointestinal infections and those caused by treatments (Sava, 1996). It resists the proliferation of tumor cells, such as human gastric cancer cells and lung fibroblasts (Guo et al., 2007); human lung and prostate cancer cells (Jabeen et al., 2017); Endothelial cells (ECV304) (Ye et al., 2008); Breast cancer cells and peripheral blood lymphocytes (Mahanta et al., 2015). Lysozyme also has anti-HIV1 capability (Varaldo et al., 1989; Yabe et al., 1993; Lee-Huang et al., 1999; Chen et al., 2018; Kawai et al., 2018).

Given the potency of lysozyme as an anti-microbial and immunomodulatory agent, it is not surprising that lysozyme has the therapeutic potential in a wide range of disease entity. In this review, we summarized the major functions and mechanisms of lysozymes. We highlighted the therapeutic applications and recent advances of lysozymes in cancers, hypertension, and viral diseases in the light of seeking alternative therapies causing no or less side effects compared to the standard medical treatment. We also explored the role of lysozyme as a cancer prognostic marker to predict patient’s outcomes and cancer recurrence probability.

### Functions and Mechanisms of Lysozyme

#### Lysozyme as an Antimicrobial Agent (Antibacterial and Anti-fungal)

**FIGURE 2:**
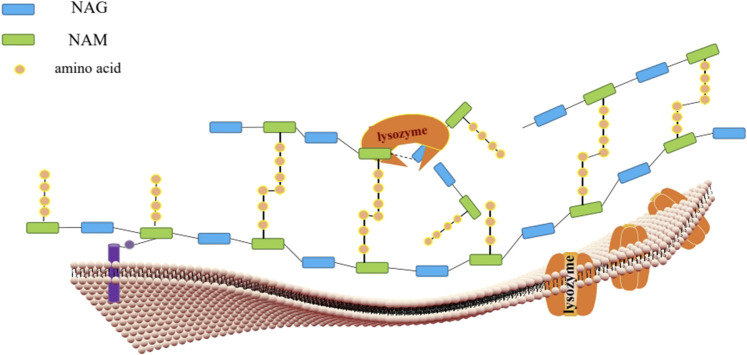
**Lysozyme is antibacterial through these two mechanisms.** The peptidoglycan(PG) skeleton of bacterial cell wall is connected by NAG and NAM through peptide stem, and then anchored on the cell membrane (purple) through lipid carrier. Lysozyme (golden yellow) hydrolyzes the interaction between NAG and NAM on PG β‐ 1,4 glycosidic bonds, leading to bacterial cell wall instability and bacterial death. Secondly, lysozyme (golden yellow) can form pores on negatively charged cell membrane by using its own cation mechanism to achieve sterilization.

Based on the enzymatic activity of lysozyme (as shown in Figure 1), it is traditionally believed that lysozyme only has antimicrobial effect on Gram (+) and has no effect on Gram (-) (Davis et al., 2008; Laaberki et al., 2011; Rae et al., 2011). This is due to the large difference in the content of peptidoglycan in Gram (+) and Gram (-) cell walls (Rae et al., 2011). Gram (+) cell wall is mainly composed of multilayer peptidoglycan and phosphoteichoic acid (Rae et al., 2011). The cell wall of Gram (-) is composed of a monolayer of peptidoglycan which does not contain teichoic acid, and is mixed between the intima and adventitia (Dillard and Hackett 2005; Iwata et al., 2016; Ragland et al., 2017). Therefore, lysozyme is considered to be more effective in killing Gram (+) (Davis et al., 2008; Laaberki et al., 2011; Rae et al., 2011). In 1991, Ibrahim et al. illustrated that the anti-bacterial effects of lysozyme on Gram (-) by studying the chemical modification of lysozyme (Ibrahim et al., 1991; Ibrahim et al., 1992; Ibrahim et al., 1994). The peptides produced by lysozyme hydrolysis have also been proved to further enhance their antibacterial effect (Mine et al., 2004). For example, the peptides corresponding to amino acid residues (aa) 15–21, 98–108 (Mine et al., 2004) and 98–112 (Pellegrini et al., 2000) have an antibacterial effect on Gram (−) such as *Escherichia coli*. The cell wall enzyme activity of lysozyme does not seem to be necessary to kill bacteria *in vitro* or *in vivo*. Then another bacteriostatic mechanism of lysozyme was found, which is a cationic antibacterial protein, lysozyme can perforate on the negatively charged bacterial cell membrane to form regular ion channels, resulting in the outflow of a large number of contents from the cells (Derde et al., 2013; Zhang et al., 2016), which eventually leads to the death of bacteria (Figure 2). Therefore, the enzymatic activity and cationic characteristics of lysozyme are the theoretical basis for lysozyme as an antibacterial (Table 2).

**TABLE 2:** The role of lysozyme in diseases.

Effect	Form	Mechanisms	Ref
Antibacterial	Intact and/or peptides	Hydrolyzes cell wall of Gram-positive bacteria (enzyme activity)	(Ibrahim et al., 1991; Ibrahim et al. 1992; Ibrahim et al. 1994; Pellegrini et al. 2000; Mine et al. 2004; Derde et al. 2013; Zhang et al. 2016)
		Insert into and form pores in negatively charged bacterial membranes	
Antifungal	Intact and/or peptides	Enzymatic activity	(Fiolka et al. 2005; Wang et al. 2005; Manikandan et al. 2015; Hernandez-Tellez et al. 2017; Sebaa et al. 2017)
		Cationic nature leading to membrane destabilization	
		Agglutination effect	
Immune modulator	Intact and/or peptides	Lysozyme in bacteria-containing phagosomes activates the pro-inflammatory responses of neutrophils and macrophages	(Li et al. 1995; Ogundele 1998; Markart et al. 2004; Liu et al. 2006; Masumoto et al. 2006; Herskovits et al. 2007; Cocchietto et al. 2008; Lee et al. 2009; Wolf et al. 2011; Caruso et al. 2014; Lemon and Weiser 2015; Muller et al. 2015; Zhang et al. 2015; Ragland and Criss 2017; Wang et al. 2017)
		Decreases chemotaxis in neutrophils	
		Suppresses TNF- α and IL-6 production by macrophages	
		Facilitates excretion of AGEs	
		Disrupts binding of peptidoglycans to complement	
		ACE inhibitory activity	
		Antioxidant activity	
Anti-cancer agent	Intact and/or peptides	Directly activate immune effectors: Tumor cell co culture increased tumor cell immunogenicity	(Babin and Babin 1973; Osserman et al. 1973; Azuma et al. 1978; Sugimoto et al. 1978; Tanaka et al. 1979; LeMarbre et al. 1981; Namba et al. 1981; Rinehart et al. 1982; Sava et al. 1985; Vacca et al. 1985; Sava et al. 1989; Varaldo et al. 1989; Yabe et al. 1993; Guo et al. 2007; Ye et al. 2008; Mahanta et al. 2015; Khan et al. 2019)
		Human lysozyme stimulated lymphocytes to proliferate in response to mitotic stimulation, and HEWL induced inhibitory and helper T cells	
		Human monocytes are activated by human lysozyme (the main secretion product of macrophages) and positive proteins similar to the cytotoxicity stage	
		(Murine fibrosarcoma; Inhibin of growh of Dalton’s lymphoma by modification of the cell surface with lysozyme; Inhibited the proliferation of endothelial cells (ECV304) and the growth of xenograft mouse sarcoid S180 and hepatoma 22 models; MCF-7 breast cancer cells; HIV-1; Ongastric cancer cell line and normal human lung fibroblasts)	
		Indirectly enhance host immunity: Lysozyme can release polyribopyrimidine acid and induce the production of interferon	
		Lysozyme cleavage activity on bacterial cell wall can release high molecular weight and low molecular weight peptidoglycan, which has been proved to have an immunomodulatory effect and antitumor activity	
		By blocking the interaction between S100A6 and RAGE (Inhibited the proliferation of SW480 (human colon adenocarcinoma cells))	
		Regulate interleukin-2 and then regulate lymphocyte proliferation. (Lymphoproliferative tumors)	
		Enzymatic activity	
		Cationic nature leading to membrane destabilization	
		Agglutination effect (Mice bearing Lewis lung carcinoma; Reduced the formation of spontaneous lung metastasis in mice with B16 melanoma; Improving the efficacy of 5-FU on primary tumor growth and lung metastasis; Disseminated tumors; Abdominal metastatic dissemination after operation of small intestinal reticulosarcoma; The prevention of graft versus host disease in patients with blood cancer undergoing a donor stem cell transplant)	
Prognostic marker for cancer	Intact	Inhibit the expression of Lysozyme, affect the cytoskeleton, down-regulate the expression of RhoA and rock, and inhibit the invasion and migration of lung cancer A549 cells	(Tahara et al. 1982; Tichy et al. 1990; Vizoso et al. 2001; Serra et al. 2002; Sun et al. 2016; Vasilescu et al. 2016)
		Lysozyme secretion may be a good prognostic marker for FBC	
		Lysozyme secretion may be a marker of poor prognosis in MBC	
		Serum lysozyme content is a marker of poor prognosis of gastric cancer	
		The content of serum lysozyme may be a marker of colon cancer	
		The content of salivary lysozyme may be a marker for monitoring the development of malignant tumors	
Lysozyme in hypertension	Intact	Enhance or limit inflammatory immune response	(Rey et al. 2001; Yoshii et al. 2001; Lewington et al. 2002; Liu et al. 2003; Janket et al. 2006; Back et al. 2007; Qvarnstrom et al. 2008; Wenzel et al. 2011; Rao et al. 2012; Labat et al. 2013; Moreno-Navarrete et al. 2021)
Antiviral	Intact and peptides	Inhibits viral entry by binding to cell receptors or virus–cationic and hydrophobic nature is required rather than enzymatic activity	(Luniakin and Bogomaz 1977; Gavrilenko et al. 1992; Steinrauf et al. 1999; Lampi et al. 2001; Lee-Huang et al. 2005; Behbahani et al. 2018; Malaczewska et al. 2019)
		Binds nucleic acids	
		Inhibits virus-induced cell fusion	
		Affects cell signaling, including the NF-κB pathway, to influence susceptibility to infection	
ACE: Angiotensin-converting enzyme; AGE: Advanced glycation end product			

In recent years, with the finding of lysozyme in different organisms, the antibacterial events of lysozyme have gradually expanded. It was observed that lysozyme, especially the ones in plants and insects, can not only resist bacteria but also fungi (Gálvez-Iriqui et al., 2020). For example, lysozyme isolated from *Pithecellobium dulce* seeds has antifungal activity against *Macrophomina phaseolina* (Gálvez-Iriqui et al., 2020). Lysozyme isolated and purified from cauliflower tissue has shown the antibacterial and antifungal capability, which can affect the growth of plant pathogenic fungi and bacteria (Manikandan et al., 2015). *Galleria mellonella* lysozyme can resist the fungus *Candida albicans* (Sowa-Jasilek et al., 2016). HEWL also has antifungal activities, such as anti-biofilm effect on *Paracoccidioides brasiliensis* (Lopera et al., 2008) and *Candida albicans* (Sebaa et al., 2017). With the advance of research, other antifungal mechanisms of lysozyme were found, in addition to enzyme activity and cationic properties. Firstly, the agglutination effect of lysozyme on the cell surface degrades the important proteins and polysaccharides in the cell wall (Gálvez-Iriqui et al., 2020). Secondly, the apoptosis of *Candida albicans* caused by lysozyme is attributed to the loss of mitochondrial membrane potential, the exposure of phosphatidylserine in the outer leaflets of the cell membrane, chromatin condensation and DNA breakage, but the mechanism has not been fully understood (Sowa-Jasilek et al., 2016). In addition, the effect of lysozyme on the fungal structure may be related to the production of β-1,3-glucanase (Hernandez-Tellez et al., 2017). The inhibitory mechanism of lysozyme on fungal and yeast polysaccharides is not completely clear. The current reports are summarized in Table 2.

Although research on the antifungal mechanism of lysozyme is not complete, the antibacterial and antifungal activities of lysozyme are affirmed. The enhancement of lysozyme as antibacterial and antifungal agents has been reported (Wu et al., 2018). Lysozyme isolated and purified from the pupa of *Cameraria ohridella* not only has bacteriostatic effects on the digestive pathway, which can decompose the bacteria ingested in the intestine, but also has a defensive response to the pathogens entering the hemocoel (Fiolka et al., 2005). The lysozyme purified from mung bean further confirmed this result (Wang et al., 2005). Other studies have shown that the antifungal activity of human lysozyme against common bacteria in patients with chronic rhinosinusitis is more than 80%, including *Aspergillus fumigatus*, Penicillium sp., Acremonium sp., *Candida parasilopsis*, and *C. albicans* (Manikandan et al., 2015). Chitosan and lysozyme coating had a good preservation effect on the quality of large yellow croaker (*Larimichthys crocea*) during cold storage with no toxic and side effects (Wu et al., 2018). In conclusion, it is necessary to first understand how enzymes act on fungi and yeast to realize their technical applications in medicine, food, and the agricultural industry.

#### Lysozyme as an Immune Modulator

The immunomodulatory function of lysozyme has only recently been paid attention to (Ragland and Criss, 2017), although it has been reported for a long time that lysozyme is an important part of biological innate immunity (Callewaert and Michiels 2010; Lelouard et al., 2010). Lysozyme fights against bacterial and fungi as a member of the immune system (Pipe, 1990; Callewaert and Michiels 2010). It was proved that the lack of lysozyme M and P significantly increase the susceptibility to respiratory tract infection, especially the infection related to the colonization of Gram (+) (Markart et al., 2004). The strong evidence showed that lysozyme participates in innate immunity. Research evidence shows that lysozyme plays an important role not only in defense mechanism but also in regulating immune response by promoting inflammatory immune response and limiting inflammatory response (Ganz et al., 2003; Nash et al., 2006; Rae et al., 2011; Ragland et al., 2017).

#### Lysozyme Stimulates the Pro-Inflammatory Immune Rsponse

Stephanie et al. revealed that the immune activation of other phagocytes may be regulated by lysozyme in the study of the relationship between the inflammatory response promoted by *Neisseria gonorrhoeae* (GC) and lysozyme and human neutrophils (Ragland et al., 2017). More studies supported this view. The ability of lysozyme to hydrolyze PG (peptidoglycan) directly affects the production of NODs (nucleotide-binding oligomerization domain, NOD) recognition receptors, and NODs can activate NF- κB senses PG, stimulating downstream pro-inflammatory signaling events, and then affect the inflammatory response (Masumoto et al., 2006; Caruso et al., 2014). Pattern recognition receptors activated downstream of lysozyme-mediated degradation also include toll-like receptors (TLRs) and inflammatory bodies. For example, the receptors TLR2 and TLR9 of bacterial derived lipoproteins and DNA were associated with the production and increase of inflammatory cytokines, such as TNF-α and IL-6, and lysozyme mediated phagosome, when phagocytes degrade *Staphylococcus aureus*, was related to the increase of the above inflammatory cytokines (Wolf et al., 2011). In addition, lysozyme in macrophages released a large amount of pathogen-associated molecular pattern (PAMP) when degrading bacterial PG, stimulated strong pro-inflammatory cytokine response and activated inflammatory bodies, such as TNF-α, IL-6, IL-12 and IFN, and enhance the activities of neutrophils (Herskovits et al., 2007; Lemon and Weiser, 2015; Muller et al., 2015). The degradation of PG by extracellular lysozyme limited the activation and recruitment of phagocytes (Herskovits et al., 2007; Lemon and Weiser 2015; Muller et al., 2015).

#### Lysozyme Limits the Inflammatory Response

In the early stage of diabetes, oral intake of lysozyme significantly decreases advanced glycation end products (AGE) concentration in serum and its deposition in the kidneys, which prevented the occurrence of microalbuminuria, buffered the proinflammatory role of AGEs, and protected the kidneys (Cocchietto et al., 2008). Zhang et al. developed a murine model of Crohn’s disease lysozyme to inhibit intestinal inflammation (Zhang et al., 2015). Lysozyme P depends on receptors NOD2 and RIP2. However, intestinal inflammation and Paneth cells failed to be classified and related to the secretion of lysozyme P (Wang et al., 2017). It was also reported that the intestinal inflammation in mice with dextran sodium sulfate-induced colitis was improved treated with lysozyme (Lee et al., 2009). The antioxidant event of lysozymes made it immunosuppressive as an exogenous additive. The addition of lysozyme reduces the chemotaxis and oxidative burst of neutrophils (Gordon et al., 1979; Ogundele 1998). Direct binding and neutralization of extracellular Pro oxidative bioactive derivatives inhibit the inflammatory response because these derivatives are advanced glycation end products and promote the inflammatory response (Li et al., 1995; Liu et al., 2006).

In conclusion, lysozyme, as an immunomodulator, enhances or inhibits the immune response. Table 2 summarizes the mechanism of lysozyme enhancing or inhibiting immune response.

## Prognostic and Therapeutic Applications of Lysozymes

### Lysozyme in Cancer

#### Lysozyme as an Anti-Cancer Agent

Many experimental studies on tumor cells have confirmed that lysozyme can inhibit the proliferation of tumor cells. Yabe et al. found that it can inhibit lymphoproliferative tumors by regulating interleukin-2 and then regulating the proliferation of lymphocytes (Yabe et al., 1993). Wang et al. further demonstrated that lysozyme extracted from marine bacteria specifically inhibited the proliferation of endothelial cells (ECV304) and the growth of xenograft mouse sarcoid S180 and hepatoma 22 models in a dose-dependent manner, bypassing toxicity (Ye et al., 2008). Mahanta et al. described the inhibitory effect of self-assembled nanostructured lysozyme (snLYZ) on MCF-7 breast cancer cells, by up to 95% at 24 h (Mahanta et al., 2015). In addition, we also found that lysozymes extracted from egg white, breast milk, and human neutrophils could protect resist HIV type 1 (HIV-1) infection, which provides a new vision for the treatment of HIV-1 infection (Lee-Huang et al., 1999). Similarly, the antitumor properties of lysozyme have been confirmed in animal model experiments (Table 3). Oral intake of HEWL significantly reduced the formation of spontaneous lung metastasis in mice with B16 melanoma (Sava 1989). Oral lysozyme can also reduce the metastasis of *Lewis* in mouse lung carcinoma (Sava et al., 1991), improving the efficacy of 5-FU on primary tumor growth and lung metastasis (Sava et al., 1995). Injection of lysozyme slightly inhibited the growth of mouse hybridoma (C57Bl/6J × DBA2) (Shcherbakova et al., 2002). In animal models, lysozyme showed different levels of activity for different tumors (Sava et al., 1989). In particular, lysozyme could interfere with the development of disseminated tumors (Sava et al., 1989). Moreover, normal mice taking lysozyme were less likely to develop cancer (Das et al., 1992). Lysozyme delivery, dose and efficiency in diseases are summarized in Table 3.

**TABLE 3:** The delivery, dose and efficacy of lysozyme in diseases.

Diseases	Treatment protocol	Treatment outcome	Ref
Chemically induced tumors (mice model)	Tumor cells of a 3-methylcholantrene induced tumor inoculated into mice immunized with the same cells treated with HLZ and lethally irradiated	Immunization of mice is successful against tumor development in 42–44% of treatments	Warren et al. (1981)
mice bearing Lewis lung carcinoma	Oral administration of 100 mg/kg/day of lysozyme chloride (Lysozyme was administered to mice by supplying the daily amount of lysozyme with the powdered food)	Lysozyme treatment reduces lung metastasis development, by significantly reducing the number of metastases of large dimension (diameters greater than 2 mm) and by causing a significant increase of the percentage of animals free of large metastases, as compared with untreated controls	Sava et al. (1991)
Ehrlich-Ascites-Tumor (EAT; mice model)	Peritumoral LZ (8 mg/kg/day)	Tumor cell death and inhibition of DNA synthesis in tumor cells	Choné and Müller, (1968)
Adenocarcinoma	LZ treatment of a rat Adenocarcinoma	Inhibition of tumor growth and increase of life-span	Sava et al. (1989)
Metastasizing animal tumors (animal model)	LZ to mouse MH134 tumor or to MethA mouse tumor	Inhibition of neoplastic growth of MethA tumors	Fukawa et al. (1982)
	Intravenous HEWL (50–200 mg/kg/day) on days 1, 5, 10, 15 from intramuscular or intravenous implantation of Lewis lung carcinoma or mammary carcinoma of CBA mouse	50% reduction of primary tumors: 35–50% and 60–70% reduction of number and weight of metastases, respectively	Sava et al. (1986)
	Oral HEWL (12.5–400 mg/kg/day) from tumor implantation to termination (and shorter treatment) in mice with Lewis lung carcinoma	60% reduction of metastasis weight with 25 mg/kg/day independently of the length of treatment; same action with treatments before tumor implantation	Sava et al. (1988b)
	Oral HEWL (35 mg/kg/day) after surgical removal of primary Lewis lung carcinoma tumors	50% reduction of lung metastases and significant increase of life-span	Sava et al. (1988b)
	Oral ELZ (25–100 mg/kg/day for 7days), or plasma and peritoncal resident cells from lysozyme treated mice to mice bearing mammary carcinoma of CBA mouse mammary carcinoma	Significant inhibition of metastatic tumor to about 50% of controls with each treatment performed	Sava et al. (1988a)
BI6 melanoma cells (mice model)	Oral HEWL (50 mg/kg/day) to BD_2_F_1_ mice on days 1–7 after the intramuscular implantation of 10^6^ B16 melanoma cells	Significant reduction in the development of lung metastases as compared with that in untreated mice	Sava (1989)
Lymphocytoma (mice model)	Intravenous HEWL (100 mg/kg/day) at mice C57Bl/6J with the transplanted ascitic	Significantly potentiates antitumor activity of cyclophosphamide, though it had no effect on the rate of tumor growth	Shcherbakova et al. (2002)
Post-transfusion hepatitis	Intravenous lysozyme chloride 60–170 mg/day	Reduced the incidence of hepatitis after transfusion from 20% to 8%	Sato et al. (1981)
Chronic crural ulcerations refractory	Local treatment with a solution of ovalbumin lysozyme in normal saline (solution in 0.9% NaCl, 1 mg/ml)	The ulcerations were cleared quickly of pus, granulation tissue developed, the inflammatory reaction around the ulcers decreased and pains were no longer felt	(Gasior-Chrzan 1988; Artym and Zimecki 2013)
Abbreviations: LZ = lysozyme. HCL of unspecifid origin; HEWL = Hen egg-white lysozyme			

Scientists have verified the inhibitory effect of lysozyme on tumors through tumor cell mixing, peritumoral and intratumoral treatment, systemic injection, oral treatment, indirect administration, or combination with other drugs (Sava et al., 1989; Khan et al., 2019). What is the anti-tumor mechanism of lysozyme? Most of the researches on the antitumor effects of lysozyme established *in vitro* studies. Lysozyme can release polyribopyrimidine acid and induce the production of interferon (Babin and Babin, 1973). The peptidoglycan fragment hydrolyzed by lysozyme has antitumor activity (Azuma et al., 1978). MD. Imran Khan has developed a profound study on the mechanism showing that lysozyme inhibited the proliferation of SW480 cancer cells, and it was found that lysozyme has the potential to inhibit cell proliferation by blocking the interaction between S100A6 (originally known as calcium cyclin, a calcium-binding protein belonging to S100 family) and RAGE (Receptor for Advanced Glycation Endproducts) (Khan et al., 2019). Lysozyme naturally secreted by monocytes and macrophages, might interact with receptor sites on the surface of lymphocytes and participate in complex monocyte-phagocyte-lymphocyte interaction and the regulation of lymphocyte activation (Varaldo et al., 1989). Lysozyme also inhibits lymphoproliferative tumors by regulating interleukin-2 (Yabe et al., 1993). The anti-tumor mechanism of lysozyme can be summarized as direct activation of immune effectors and indirect enhancement of host immunity (Table 2).

Based on the antitumor properties of lysozyme, lysozyme is highly expected to be used as an anticancer agent (Khan et al., 2019). For a long time, the treatment of tumors mainly depended largely on radiotherapy and chemotherapy (Furue, 2003). Although the effect is remarkable, it still has strong toxic and side effects, and new anti-tumor therapy is urgently needed (Xia et al., 2003). Guo et al. purified recombinant human lysozyme (rhlys) through traditional molecular cloning technology and tested the anti-proliferation effect on gastric cancer cell line and normal human lung fibroblasts (Guo et al., 2007). It was found that lysozyme at a concentration of 100 μg/L could effectively inhibit the growth of cancer cells without showing any toxicity on normal cells (Guo et al., 2007). Experiments showed that lysozyme as a natural anticancer drug, compared with antibiotics and other drugs, had no toxic or side effects (Lee-Huang et al., 1999). In as early as 1964, lysozyme was reported to be used to treat cancer. *Laterza* injected lysozyme into a patient with abdominal metastatic dissemination after surgery of small intestinal reticulosarcoma. When the lysozyme injection was measured to 45 g lyophilized powder and the patient’s abdominal metastasis disappeared (Sava et al., 1989). In the follow-up investigation, the patient was still healthy 6 years later (Sava et al., 1989). In 1978, lysozyme successfully authorized a patent in Japan for the treatment of cancer, and it was proposed that lysozyme oral agent can strengthen the immunity of cancer patients (Sava et al., 1989). At present, there are many clinical trials of lysozyme drugs under research (Airapetova et al., 2013). As an anti-cancer agent, human lysozyme goat milk for the prevention of graft versus host disease in patients with blood cancer undergoing a donor stem cell transplant. It has been used in combination and is in phase 1 of clinical trial (ClinicalTrials.gov NCT04177004). Lysozyme as an anticancer agent may be a new treatment option for cancer (Zhivotovsky and Orrenius 2010).

#### Prognostic Application of Lysozymes

##### Lysozyme as a Prognostic Marker for Cancer

The challenge of cancer treatment is due to not only the malignant value-added of the tumor but also its high clinical variability (Du et al., 2009). Therefore, exploring new prognostic biomarkers, aiming to understand the pathophysiological development of cancer and facilitate treatment (Khurshid et al., 2018). Lysozyme can not only be regarded as a drug candidate for cancer treatment but also be considered as a promising biomarker for the early diagnosis, staging and prognosis of tumors (Wang et al., 2016). Based on Cui huaibo’s (Vasilescu et al., 2016) finding, lysozyme was related to the invasion of lung cancer cells. Wang et al. further explored the mechanisms of lysozyme on the invasion and migration of lung cancer cells A549 (Wang et al., 2016). The study found that lysozyme mediated the invasion and migration of A549 cells by activating Rho family proteins of related cytoskeleton signaling pathways, which suggests that lysozyme may be a potential protein marker for the progression and prognosis of lung cancer (Wang et al., 2016). Vizoso et al. found that the expression of lysozyme predicted the rate of recurrence-free survival and the rate of overall survival of lymph node-negative patients in FBC (female breast cancer), which supports that lysozyme is a prognostic marker for the benign development of FBC tumors (Vizoso et al., 2001). However, in the study of male breast cancer (MBC), opposite results were obtained. Although lysozyme can also be used as a prognostic marker protein of MBC, studies have found that the expression of lysozyme in MBC patients was related to the poor prognosis of cancer (Serra et al., 2002). This may be caused by different gender and different hormone secretion levels. Lysozyme secretion can be detected in 15% of normal epithelium next to female breast tumors (Vizoso et al., 2001), but it is not detected in male breast development patients. It is found that the increased expression of lysozyme in gastric cancer is related to the poor prognosis of patients (Tahara et al., 1982). The 2-years poor survival rate of gastric cancer patients implied the poor prognosis of advanced gastric cancer containing lysozyme (Tahara et al., 1982). The growth rate, invasion and metastasis of gastrointestinal malignant tumors are related to the levels of lysozyme in serum, which can be used as a marker of colorectal cancer patients (Tichy et al., 1990). The content of lysozyme in saliva can also reflect the process of malignant tumors to a certain extent. The later stage of tumor development affects the immune ability of patients and then affects the secretion level of lysozyme protein in the mouth (Sun et al., 2016). Based on its regulatory effects on the human immune system, lysozyme has the potential to be a prognostic marker in the initiation, development, as well as metastasis of most cancers (Table 2).

##### Lysozyme as a Marker in Other Diseases

With further researches, lysozyme as a biomarker to monitor the occurrence and development of diseases is not only in the field of cancers. Compared with normal tissues, the levels of lysozyme in Barrett’s esophageal columnar epithelial cells were significantly increased, suggesting that lysozyme may be involved in the formation of Barrett’s esophagus (Serra et al., 2002). MC colonial colitis (CC) or lymphocytic colitis (LC) is caused by bacterial invasion. Therefore, the patients with these two diseases will be accompanied by the increase of lysozyme (Rubio, 2011). Lysozyme is an effective marker to monitor the two diseases. In the study of the relationship between autoantibodies against non-myeloperoxidase (MPO) neutrophil particle antigen and Behcet’s disease (BD) activity, only anti-lysozyme was significantly correlated with BD disease activity, which was the only independent marker for predicting active disease in BD patients (Park et al., 2017). Schroeder pointed out that lysozyme is a marker for the diagnosis and progression of myocarditis (Schroeder et al., 2000). In the study of tears as a minimally invasive biological fluid, it was found that lactoferrin and lysozyme have the potential to be biomarkers of mucosal immunity (Hanstock et al., 2019). In addition, the expression of lysozyme is helpful to distinguish acinic cell carcinoma (ACC) from its main mimic, mammary analog secretory carcinoma (MASC), because the latter has a higher frequency, while ACC does not exist (Park et al., 2017). In conclusion, lysozyme can be used as a marker of a variety of diseases, which is of great research value.

### Lysozyme in Hypertension

Hypertension is a disease with a high prevalence worldwide. Hypertension is the main risk factor of atherosclerosis, coronary heart disease, stroke, chronic kidney disease, and heart failure (Lewington et al., 2002). About 95% of patients with hypertension are primary hypertension, but its etiology is not clear (Qvarnstrom et al., 2008). Vascular endothelial dysfunction caused by chronic inflammation or impaired glucose metabolism may be related to it (Reaven, 2002; Bautista, 2003; Grundy, 2003; Janket et al., 2006). As mentioned above, lysozyme can stimulate pro-inflammatory immune responses and inhibit the inflammatory response. It is speculated that lysozyme may affect hypertension. This conjecture was confirmed by subsequent studies. Researchers have found that increased lysozyme content in the saliva is an indicator of the early stage of hypertension (Janket et al., 2006). Logistic regression analysis of 500 Finnish people with or without coronary heart disease in the Kuopio oral health and heart study showed that people with higher levels of lysozyme were more likely to suffer from hypertension (Qvarnstrom et al., 2008). Moreover, obesity is an important risk factor for hypertension (Doll et al., 2002). Increased plasma lysozyme levels and activity are found in obese subjects, the plasma lysozyme might be protective on the development of obesity-associated metabolic disturbances (Moreno-Navarrete et al., 2021). It is indirectly proved that the increase of lysozyme is an early index of hypertension. Angiotensin II (ATII) is an effective vasoconstrictor, which can cause hypertension (Rey et al., 2001; Liu et al., 2003). It was found that the infiltration of monocytes- and macrophages-secreting lysozyme may be an important factor in ATII-induced vascular dysfunction and arterial hypertension (Wenzel et al., 2011). Similarly, in the study of HEWL, Rao et al. reported that HEWL inhibited the development of hypertension in spontaneously hypertensive rats by inhibiting angiotensin-converting enzyme (ACE) (Yoshii et al., 2001; Rao et al., 2012). Carlos Labat can predict cardiovascular disease and risk factors from the inflammatory mediators in saliva (Labat et al., 2013), and lysozyme is the main inflammatory mediator in saliva (Janket et al., 2006; Back et al., 2007). In conclusion, indirect and direct evidence shows that lysozyme is an index to predict hypertension and other related diseases by regulating inflammatory immune response (Table 2).

### Lysozyme as an Antiviral Agent

Since the discovery of autolysozyme, people have been exploring the possibility of the antiviral capability of lysozyme. In the late 1950’s, the papers from the first, second and third Fleming lysozyme International Symposium held in Milan mentioned that lysozyme was used to treat several human viruses and achieved positive results. The antiviral activity of lysozyme was officially valued by the scientific research community (Satta, 1984). Zhang et al. studied the antiviral activity of lysozyme extracted from marine bacterium S-12–86 and found that it has a strong inhibitory effect on rabies virus (PRV) (Zhang et al., 2008). More studies showed that lysozyme was anti-adenovirus and can be used to treat herpes, mumps, chickenpox, hepatitis, influenza, and atypical pneumonia (Zhang et al., 2008). In the study of lysozyme against herpesvirus, by improving the thermal activation of HEWL, it was found that lysozyme depended on its cationic protein characteristics rather than enzyme activity (Satta, 1984). And lysozyme inhibits virus entry by binding with cell receptor or virus (Lampi et al., 2001), binding nucleic acid (Lee-Huang et al., 2005), and inhibiting virus-induced cell fusion; The antiviral mechanisms that affect cell signals, including NF-κB pathway and infection susceptibility (Steinrauf et al., 1999; Lampi et al., 2001; Lee-Huang et al., 2005; Behbahani et al., 2018) are summarized in Table 2. Many researchers have proposed that the antiviral properties of lysozyme can be used to treat coronavirus disease (COVID-19) (Mann, 2020), because the immune response that can be regulated by lysozyme (Table 2) is consistent with the typical characteristics of severe COVID-19, such as oxidative stress, inflammation caused by neutrophils, macrophages, TNF- α, and IL-6, and activated RAS system (Jomova and Valko 2011; Chen et al., 2020; McGonagle et al., 2020; Mehta et al., 2020; Park, 2020; Shoenfeld, 2020; Zhou et al., 2020). In Eastern Europe, lysozyme has been used in combination with antibiotics to fight bronchitis and pneumonia without showing cytotoxicity (Luniakin and Bogomaz, 1977; Gavrilenko et al., 1992). Oral lysozyme administration is a new medical therapy to enhance the immunity of the organism and then fight against viral infection. Oral administration of HEWL at 1 g/day resisted herpes (Sava, 1996). More studies have used the coadministration of lysozyme and lactoferrin in the treatment of bovine viral diarrhea virus, which was more effective than lysozyme or lactoferrin alone, and lysozyme will not weaken the drug efficacy over time (Malaczewska et al., 2019).

In summary, several main challenges of using lysozyme as a candidate drug that should be addressed in future research: 1) The mechanisms of lysozyme anticancer and disease markers are not clear, and the theoretical basis is incomplete; 2) There are few clinical trials of lysozyme as a drug currently; 3) At present, the research of lysozyme as a drug is mainly focusing on human lysozyme and HEWL, without in-depth exploration in other types; 4) Human lysozyme has a high production cost (depending on the process), low operational stability, solvent inactivation, and a lack of recovery or recycling. Future investigations to address these issues will be valuable.

## Future Directions

This review mainly introduced the antibacterial, antifungal, and antiviral mechanisms of lysozyme, as well as its application in diseases such as cancer and hypertension. This is based on the powerful function of lysozyme itself, including lysozyme in bacteria-containing phagosomes activates the pro-inflammatory response of neutrophils and macrophages (Tagashira et al., 2018); reduces the chemotaxis of neutrophils; inhibits the production of TNF- α and IL-6 by macrophages, promotes the excretion of AGEs, etc. as listed in Table 2. As a natural preparation, lysozyme has the advantages of good tolerance and no side effects. The medical application of lysozyme is not limited to what has been mentioned above. For example, it has been reported that the lysozyme chloride at the dose of 60–170 mg for 4–24 weeks reduced the incidence of hepatitis after transfusion from 20 to 8% (Sato et al., 1981). Ovalbumin lysozyme normal saline solution was used to treat chronic leg ulcers in patients who have failed to respond to previous treatment (Gasior-Chrzan 1988). After adding 50 mg/L lysozyme to the milk for premature infants who had the disease for 2–3 weeks, the inflammatory focus in feces disappeared rapidly (Bol’shakova et al., 1984; Artym and Zimecki, 2013). The combinational dosing of human lysozyme and milk protein reduced intestinal dysfunction in Malawian children (Cheng et al., 2019). There are few clinical achievements of lysozyme in the human body, and the clinical administration of lysozyme depends more on experience (Sava, 1996; Artym and Zimecki, 2013). Existing literature provides evidence on the promising use of lysozyme in human diseases. However, it is largely based on case reports or local studies with a small number of subjects, which cannot be generalized to the global population, and limited by a lack of follow-up data to support the long-term benefits or show side effects. The synergistic effect of lysozyme with other dietary supplements should be tested further to confirm the beneficial effects, as well as the optimal dosing, timing, and duration.

In addition to the medical application, lysozyme is widely used in the food and cosmetics industries. Especially in food safety, it is a public problem that the world pays more and more attention to food safety (Sheveleva et al., 2020). Lysozyme can be used for food preservation such as cheese production through its antibacterial activity (Naidu, 2000) and beer preservation by delaying the growth of spoilage bacteria without high-temperature sterilization (Makki and Durance, 1996). However, it remains challenging to put lysozyme into practical usage due to high cost (depending on the process), low operational stability, solvent inactivation, lack of recovery or recycling and so on (Gálvez-Iriqui et al., 2020). Therefore, immobilizing enzymes in the polymer matrix is one of the main research focuses of lysozyme, which aims to prolong its activity and effectiveness in the food industry (Yu et al., 2018). Chitosan is a polymer showing promising results in experiments (Yu et al., 2018).

At present, the most commercialized lysozyme is HEWL as the structure of HEWL is similar to human lysozyme. However, its potency is much weaker than human lysozyme, and the recovery cost is high (Ercan and Demirci, 2016). The most important thing is that people with egg white allergy will secret specific IgE antibody titer when this lysozyme is used (Naidu, 2000). Therefore, the industrial production of human lysozyme is a technical difficulty to be overcome. Although many studies have used transgenic plants and animals to produce human lysozyme. For instance, somatic cell-mediated transgenic cloning was used to produce recombinant human lysozyme in the milk of transgenic goats (Yu et al., 2013), the expression of human lysozyme in the milk of transgenic mice (Yu et al., 2006), the transgenic carrot produced by human lysozyme and the expression (Mattanovich et al., 2012) of human lysozyme gene in rice (Wilken and Nikolov, 2011). However, its advantages are not as good as microorganisms that have a high growth rate, simple growth conditions, and low cost (Mattanovich et al., 2012). However, microbial fermentation still faces the challenge of many technical parameters, such as the selection of fermentation strains and reactors, and the optimization of pH value, temperature and dissolved oxygen (Yang et al., 2011; Yu et al., 2013). The selection of engineered fermentation strains has been reported in 2004. Choi et al. produced human lysozyme through *S. cerevisiae* fermentation. *S. cerevisiae* has intracellular retention and high glycosylation of secreted proteins (Choi et al., 2004). In-depth study found that *Kluyveromyces lactis* is more suitable for the production of recombinant human lysozyme (Rossolini et al., 1992; Iwata et al., 2004). Compared with *S. cerevisiae*, *K. lactis K7* has the highest lysozyme yield and genetic stability in large-scale production (Rossolini et al., 1992). In the selection of reactors, it was found that a biofilm reactor (173 U/ml) improved the yield of lysozyme more than a suspension cell reactor (110 U/ml) (Ercan and Demirci, 2016). In addition, the separation of lysozyme is also a technical challenge (Chia et al., 2019). Lysozyme is an active protease. Researchers need to purify it quickly without affecting its enzyme activity to produce a high yield (Ercan and Demirci, 2016). Lysozyme contains antimicrobial peptides, which may affect the growth of cells in the fermentation process. Therefore, synchronous fermentation and an online recovery system are used to further improve the yield of lysozyme to 280.4 U/ml (Ercan and Demirci, 2016). The online recovery system is also changed to macroporous resin KA-I as adsorbent. Compared with traditional batch fermentation, the solvent concentration and productivity were increased 4 to 6 fold, respectively (Liu et al., 2014). Through these improvements, it is expected that the industrial production of recombinant human lysozyme will be achieved in the near future.

In the past, researchers had focused mostly on lysozyme in terrestrial microbes. The rapid changes of diseases have occurred without warning. Diseases such as SARS and COVID-19 (Mann, 2020) are not sensitive to any existing drugs. Researchers should pay more attention to marine organisms. For example, Japan and the United States have reported that several antibacterial and antiviral substances have been screened from marine microorganisms for clinical application (Zhang et al., 2008). At present, many lysozymes in marine organisms have been found, and even the antibacterial spectrum was higher than that of animal and plant lysozymes (Zhang et al., 2008), which needs to be further investigated.

## Conclusion

This paper summarized the major functions and mechanisms of lysozyme, as well as the latest advances and challenges in its application. We emphasized the prognostic and therapeutic application of lysozyme in cancer, hypertension, and viral diseases. However, clinical investigation of lysozyme in human diseases is limited by a lack of follow-up data to support the long-term benefits or show side effects. Despite many studies that have been focused on the interaction between lysozyme and other proteins and signaling pathways, the mechanism remains to be elucidated. Moreover, research on the medical application of lysozyme focuses mainly on animal c-type lysozyme, while lysozyme derived from other sources such as plants, insects, and marine microorganisms showing similar antibacterial properties should also be taken into consideration in future studies. Together, the clinical investigation of lysozyme in the treatment of cancer, hypertension, viral diseases, and others may provide novel insights into alternative therapies and the prognostic potential to predict patient’s outcomes and cancer recurrence probability.
